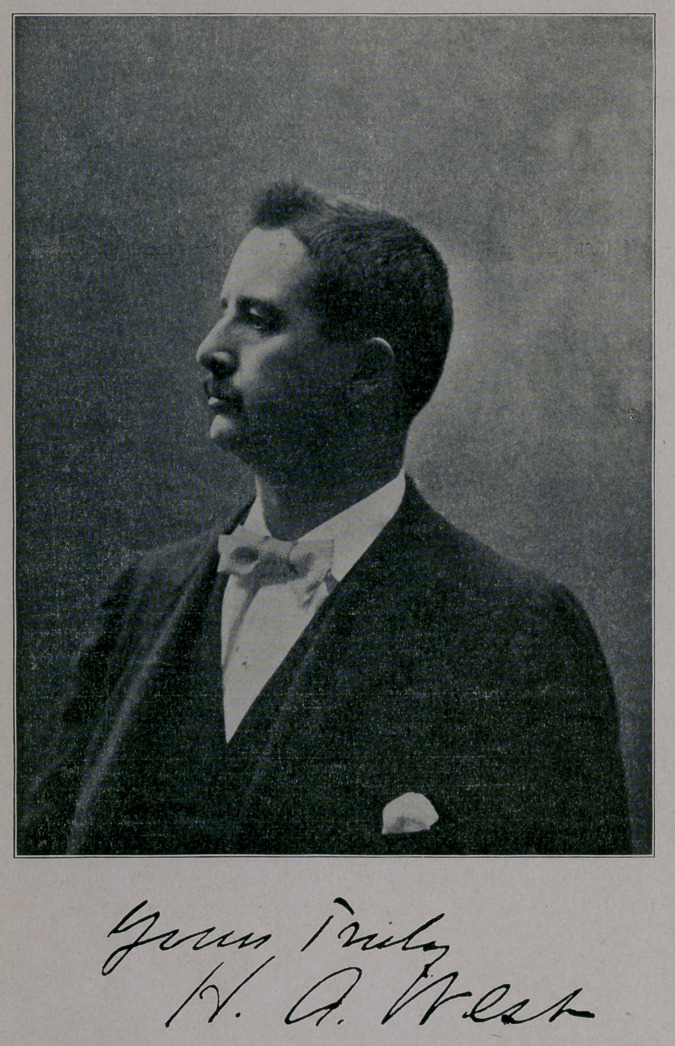# Dr. H. A. West, Secretary of the Texas State Medical Association

**Published:** 1900-06

**Authors:** 


### DR. H. A. WEST, ✓—

### SECRETARY OF THE TEXAS STATE MEDICAL ASSOCIATION.

The JOURNAL presents herewith an excellent picture of the popular Secretary of the Texas State Medical Association, Dr. H. A. West, and give below a brief sketch of his life. The doctor is the most euthusiastic medical society man we have, and goes into society work with great zeal, always contributing a large share to the interest of the meetings. He is also president of the South Texas Medical Society.

HAMILTON ATCHISON WEST, second child and eldest son of James N. and Isabella Atchison West, was born at Russell's Cave, Fayette county, Kentucky, March 30, 1849. Received a common school education in the county schools of the neighborhood. Was graduated from the medical department University of Louisville in 1872, taking the highest honors of his class—the faculty medal—for the best thesis, the subject of which was the "Thermometry of Disease." By competitive examination the following spring, was elected one of the house surgeons of the Louisville City Hospital. In the fall of 1873 came to Texas and located in Galveston, serving two years as house surgeon of the Galveston City Hospital. Took an active part in the effort which resulted in the location of the medical branch of the University in Galveston. Upon the organization of the school was elected to fill the chair of general and clinical medicine, which he occupied until his resignation, August, 1897. He joined the State Medical Association in 1887, and has read a paper at every subsequent meeting. Was elected Secretary at Waco in 1891, re-elected at Dallas in 1895 and at Waco in 1900. Was elected one of the vice-presidents of the American Medical Association at the meeting in Denver in 1898. Was one of the contributors to the American System of Medicine, writing the articles upon "Dysentery and Dengue." Contributed also to Gould & Pyles' Cyclopedia of Medicine and Surgery, writing the article upon yellow fever.

## Figures and Tables

**Figure f1:**